# Use of the Microheterogeneous Model to Assess the Applicability of Ion-Exchange Membranes in the Process of Generating Electricity from a Concentration Gradient

**DOI:** 10.3390/membranes11060406

**Published:** 2021-05-28

**Authors:** Denis Davydov, Elena Nosova, Sergey Loza, Aslan Achoh, Alexander Korzhov, Mikhail Sharafan, Stanislav Melnikov

**Affiliations:** Physical Chemistry Department, Faculty of Chemistry and High Technologies, Kuban State University, 350040 Krasnodar, Russia; davyd.denis00@mail.ru (D.D.); firofran@mail.ru (E.N.); s_loza@mail.ru (S.L.); achoh-aslan@mail.ru (A.A.); shtrih_ooo@mail.ru (A.K.); shafron80@mail.ru (M.S.)

**Keywords:** reverse electrodialysis, ion-exchange membrane, conductivity, diffusion permeability, microheterogeneous model

## Abstract

The paper shows the possibility of using a microheterogeneous model to estimate the transport numbers of counterions through ion-exchange membranes. It is possible to calculate the open-circuit potential and power density of the reverse electrodialyzer using the data obtained. Eight samples of heterogeneous ion-exchange membranes were studied, two samples for each of the following types of membranes: Ralex CM, Ralex AMH, MK-40, and MA-41. Samples in each pair differed in the year of production and storage conditions. In the work, these samples were named “batch 1” and “batch 2”. According to the microheterogeneous model, to calculate the transport numbers of counterions, it is necessary to use the concentration dependence of the electrical conductivity and diffusion permeability. The electrolyte used was a sodium chloride solution with a concentration range corresponding to the conditional composition of river water and the salinity of the Black Sea. During the research, it was found that samples of Ralex membranes of different batches have similar characteristics over the entire range of investigated concentrations. The calculated values of the transfer numbers for membranes of different batches differ insignificantly: ±0.01 for Ralex AMH in 1 M NaCl. For MK-40 and MA-41 membranes, a significant scatter of characteristics was found, especially in concentrated solutions. As a result, in 1 M NaCl, the transport numbers differ by ±0.05 for MK-40 and ±0.1 for MA-41. The value of the open circuit potential for the Ralex membrane pair showed that the experimental values of the potential are slightly lower than the theoretical ones. At the same time, the maximum calculated power density is higher than the experimental values. The maximum power density achieved in the experiment on reverse electrodialysis was 0.22 W/m^2^, which is in good agreement with the known literature data for heterogeneous membranes. The discrepancy between the experimental and theoretical data may be the difference in the characteristics of the membranes used in the reverse electrodialysis process from the tested samples and does not consider the shadow effect of the spacer in the channels of the electrodialyzer.

## 1. Introduction

Electrodialysis is an electromembrane process designed to remove ionic impurities from aqueous solutions. In electrodialysis, ion-exchange membranes of two types are used: cation-exchange membranes permeable only for cations and anion-exchange membranes permeable only for anions. When an electric current is applied to the electrodialysis apparatus, which consists of a plurality of alternating cation-exchange and anion-exchange membranes, migration of cations occurs through the cation-exchange membranes and the migration of anions through the anion-exchange membranes. During the operation of the electrodialyzer, the concentration of ionic components in one chamber, called the desalination chamber, decreases, and in the other, called the concentration chamber, increases. The two membranes (cation-exchange and anion-exchange) and the desalination chamber and the concentration chamber are collectively called a membrane pair. The collection of all membrane pairs in an electrodialyzer is called a membrane stack.

Electrodialysis can be used in several industrial processes associated with the removal of ions from aqueous solutions [1,2], electromembrane synthesis [3], processing solutions in the agro-food industry [4], and processing highly concentrated reverse osmosis or membrane distillation effluents [5,6]. In the latter case, the generated highly concentrated effluents can also be used as a source of “blue” electricity using a process called reverse electrodialysis [7].

Reverse electrodialysis (RED) generates electricity based on the utilization of energy released when two solutions with different concentrations are mixed. When using an ion-exchange membrane, which separates concentrated and dilute electrolyte solutions, due to the diffusion of a substance through the membrane, an ion flux (electric current) occurs. At the interfaces between a dilute solution/membrane and a membrane/concentrated solution, a potential difference occurs called the Donnan potential. The sum of the two potential drops at the left and right sides of the membrane is called the membrane potential (*E_m_*). Its value is determined by the ratio of ions’ activities in solutions to the right and left of the membrane. Since each of the two membranes (cation-exchange (CEM) and anion-exchange (AEM)) has its membrane potential, the total potential drop on the membrane pair will be the sum of two membrane potentials:

(1) $$\begin{array}{l} E_{\mathrm{RED}}=N\left(E_{m}^{CEM}+E_{m}^{AEM}\right),\\ E_{m}^{\mathrm{CEM}}=\alpha^{\mathrm{CEM}}\frac{RT}{nF}\ln\frac{a_{b}^{+}}{a_{d}^{+}}=\frac{RT}{nF}\ln\frac{c_{b}^{+}\gamma_{b}^{+}}{c_{d}^{+}\gamma_{d}^{+}},\\ E_{m}^{\mathrm{AEM}}=\alpha^{\mathrm{AEM}}\frac{RT}{nF}\ln\frac{a_{b}^{-}}{a_{d}^{-}}=\frac{RT}{nF}\ln\frac{c_{b}^{-}\gamma_{b}^{-}}{c_{d}^{-}\gamma_{d}^{-}} \end{array}$$

where *N* is the number of membrane pairs; α is the permselectivity of ion-exchange membrane; *c* is the electrolyte concentration, mol/L; γ is the ion activity coefficient; lower indexes “*b*” and “*d*” denote high concentration solution (brine) and low concentration solution (diluate); *R*, *T*, and *F* are universal gas constant (8.314 J/(mol·K)), absolute temperature (K), and Faraday’s constant (96487 C/mol).

In recent years, interest in reverse electrodialysis has grown significantly, as evidenced by many reviews devoted to this topic. Tian et al. [8] considered the effect of the electrode material and the redox pair used, the most critical operating parameters (solution pumping rate, concentration of brine and dilute solutions, membrane channel geometry, etc.) were considered by Mei and Tang [9], current achievements and existing problems are disclosed by Pawlowski et al. [10], and the importance of permselectivity of ion-exchange membranes in the RED process are given by Kotoka et al. [11] and Zoungrana and Cakmakci [12].

For the first time, the possibility of transforming the concentration gradient in natural conditions (for example, at the mouth of a river flowing into the sea) was shown by Pattle in 1954 [13]. The energy density obtained in work was 0.2 W/m^2^ at 39 °C using a hydroelectric pile composed of alternating 47 CEMs and 47 AEMs.

Further development of the technology made it possible to increase the energy density. Today, the average value of the energy density is 0.94 ± 0.4 W/m^2^ when using a concentrated solution, either solution from solar ponds or effluents from desalination plants (data from review [14] were taken to calculate the average value).

The increase in power density is achieved in various ways. Researchers pay special attention to the ion-exchange membranes used and their properties [14,15,16]. One of the critical properties of membranes is their permselectivity, i.e., cation-exchange membranes’ ability to transfer only cations, and of anion-exchange membranes, only anions. Considering the imperfect selectivity of ion-exchange membranes, the equation for the open-circuit voltage (OCV) of the reverse electrodialyzer takes the form:

(2) $$E_{}^{OCV}=N\frac{RT}{nF}\ln\left(\frac{c_{b}^{}\gamma_{b}^{\pm }}{c_{d}^{}\gamma_{d}^{\pm }}\right)\left(\frac{t_{g}^{*}{}^{CEM}-t^{+}}{1-t^{+}}+\frac{t_{g}^{*}{}^{AEM}-t^{-}}{1-t^{-}}\right).$$

where $t_{g}^{*}{}^{CEM}$ and $t_{g}^{*}{}^{AEM}$ are the counterion transport numbers in the cation- and anion-exchange membranes; $t^{+}$ and $t^{-}$ are the counterion transport numbers in solution.

The open-circuit voltage is the driving force of the RED process and represents the sum of potential differences over each membrane [17].

The terms in the last parenthesis in Equation (2) represent the permselectivity of the cation-exchange and anion-exchange membranes (the first term in parentheses is $\alpha^{\mathrm{CEM}}$, and the second is $\alpha^{\mathrm{AEM}}$; counterions transport numbers ($t_{g}^{*}$) are for cation-exchange and anion-exchange membranes in the first and second case, respectively). The membranes’ permselectivity can be determined knowing the values of the counterion transport number in the membrane. Different methods can be used to determine the counterions’ transport number in the membrane; a short summary of these methods is given in Table 1.

The membrane potential method [18] is perhaps is the most widely used one. However, the transport numbers determined by this method will be “apparent”, since they do not consider the transfer of water molecules within the hydration shells of ions. To obtain the “true” value of the transport number, one can use the Scatchard equation [19]. However, its use requires the water transport number’s values, which are also difficult to determine experimentally.

The three-wire model of ion-exchanger conductivity can be used for calculation of the counterion transport number [20]. In this method, the transport of co-ions is associated with the solution transport channel in the membrane, while counterions are the only charge carriers in the mixed and gel channels of conductivity. This method tends to correctly predict counterions’ transport numbers in dilute solutions, while in concentrated solutions, it tends to lower the transport number compared with the Skachard equation.

One can calculate the “true” transport numbers across an ion-exchange membrane from the concentration dependences of electrical conductivity and diffusion permeability using the microheterogeneous model [21]. In view of the microheterogeneous model, the ion-exchange membrane is represented as a combination of two phases called “gel” and “electroneutral solution”. Integral properties of the membrane, such as electrical conductivity and diffusion permeability, are determined as the geometric mean of some electrotransport coefficients, different for the gel and electroneutral solution phases.

The aim of this work is to test the possibility of using a microheterogeneous model and data on the concentration dependence of the transfer numbers of ions for various ion-exchange membranes to calculate the open-circuit potential and select the best membrane pair for carrying out the reverse electrodialysis process.

## 2. Microheterogeneous Model

The microheterogeneous model was proposed by Gnusin et al. [22] and further developed by Nikonenko and Zabolotskiy [21], Berezina et al. [23], and Demina et al. [20]. In the frame of the model, the ion-exchanger is presented as a two-phase system, each of which possesses different transport coefficients regarding ions transport. The microheterogeneous model has found numerous applications to predict or describe properties of both heterogeneous and homogeneous membranes [21,24,25]. Below, we will briefly discuss what these phases are and how the microheterogeneous model can calculate some transport properties of an ion-exchange membrane.

**Figure 1:**
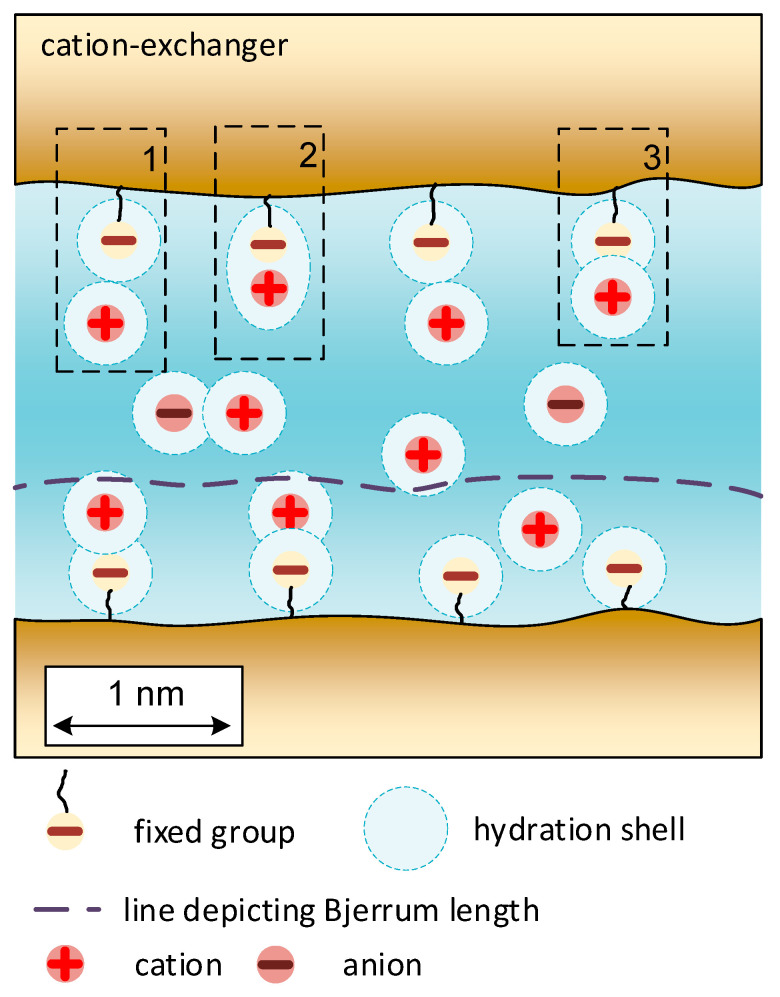
The schematic depiction of a micropore in a swelled membrane. The structure of ion’s pairs “fixed group-counterion” is shown: 1—ion pairs at the contact of undisturbed primary hydration shells, 2—inner-sphere complex, 3—outer-sphere complex.

The counterion (the ion whose charge is opposite to the charge of the fixed group) in the ion-exchange material can be in several states: a “condensed” state near the ionogenic group [26] or a “dissociated” state. An ion in a condensed state can be a part of both the inner- and outer-sphere complexes with a fixed group or form an ion pair with a fixed group with undistributed hydration shells (Figure 1). What state will be implemented depends on the amount of hydration water per the counterion and the fixed group. The greater the amount of water, the farther they are removed from each other. The dissociated state of the ion is also ambiguous. If it is in a micropore with a radius of 1.5–2.0 nm, it is constrained after dissociation. Its state is similar to the state of an “embedded” ion in a crystal. Suppose the distance at which the dissociated ion is located is close to the Bjerrum length (the distance at which the thermal energy of the ion fully compensates for the electrostatic interaction between the ion and the fixed group) [26]. In that case, the condensed and “embedded” counterions often change places. The distance between fixed groups is close to the pore radius (for Nafion membranes, distances between fixed groups are given equal to 0.6–1.2 nm [27], for membranes with a polystyrene-divinylbenzene matrix 0.5–0.7 nm [28]). Therefore, micropores with fixed ions, counterions, and hydrophilic regions of the matrix can be considered a quasi-homogeneous phase. Within the frame of the microheterogeneous model, this phase is combined with an inert binder and a reinforcing fabric (if any) and hydrophobic parts of polymer chains. The resulting phase is called the gel phase of the ion exchanger.

**Figure 2:**
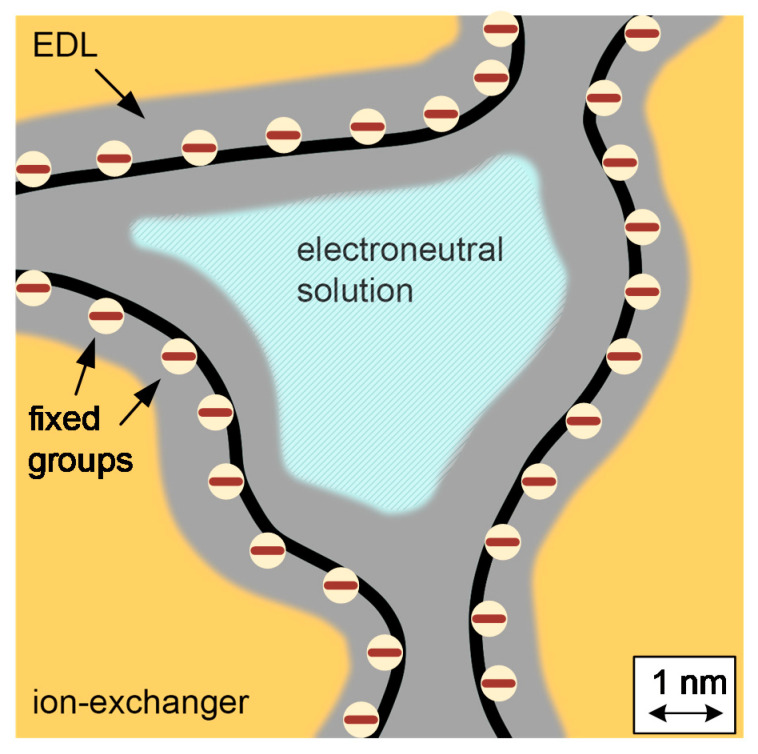
The schematic depiction of the ion-exchange membrane microstructure.

If the ion is in a sufficiently large pore with a radius > 4–5 nm, then, after dissociation, it enters an electrically neutral solution with a concentration equal to the concentration of the external solution. It is assumed that the physicochemical properties of such an ion do not differ from their properties in an external solution. This phase of an electrically neutral solution is also called an intergel solution (Figure 2).

The following ratios express the fractions of the gel and electroneutral solution phases:

(3) $$\begin{array}{l} \frac{V_{\mathrm{gel}}}{V_{\mathrm{total}}}=f_{1,}\;\frac{V_{\mathrm{sol}}}{V_{\mathrm{total}}}=f_{2,}\;\\ f_{1}+f_{2}=1 \end{array}$$

where *V*_gel_, *V*_sol_, and *V*_total_ are volumes of the gel and electroneutral solution phases, and total volume of the membrane is m^3^; *f*_1_ and *f*_2_ are the fractions of gel and electroneutral solution.

The transfer of counterions and co-ions in the ion-exchanger is carried out in different ways. The concentration of co-ions in the gel phase is relatively low, which, together with electrostatic repulsion from fixed groups, leads to low mobility. Simultaneously, according to Manning’s ion condensation theory, the counterion is located at a distance less than the Bjerrum length from a fixed group in the region of the potential energy minimum. As a result, its transfer along the ion-exchanger chains is facilitated [28].

In the phase of an electrically neutral solution, the concentration of co-ions is equal to the concentration of the external solution; also, its physicochemical properties do not differ from those of the external solution. Both factors contribute to the facilitated transport of the solute through the phase of an electroneutral solution. In ion-exchange membranes, the gel and the electrically neutral solution phases are randomly located in space, and the fraction of the electroneutral solution is 0.1–0.2 (for heterogeneous membranes) [20,25]. For homogeneous membranes, the fraction of electroneutral solution is usually less than 0.1 [24,25,29]. As was shown in [30], there are none through macropores, even in heterogeneous membranes. Thus, the transfer of ions is mainly carried out through the mixed channel: in the region of macropores through the phase of an electroneutral solution and then through the gel phase.

The equation for the ion flux through the gel phase (*J_i_*) obtained within the framework of the nonequilibrium thermodynamics has the following form [19]:

(4) $$J_{i}=-P^{*}\frac{dc_{i}}{dx}+\frac{it_{i}^{*}}{z_{i}F},$$

where $P^{*}$ is the differential coefficient of diffusion permeability, m^2^/s; *i* is the current density, A/m^2^; $t_{i}^{*}$ is the transport number of ion *i* in the membrane.

Electromigration transport numbers of counter and co-ions can be calculated based on the data on the diffusion permeability and electrical conductivity of an ion-exchange membrane [21]. For a 1:1 electrolyte, the counterion transport number ($t_{g}^{*}$) is defined as:

(5) $$t_{g}^{*}=\frac{L_{g}}{L_{g}+L_{co}},$$

where $L_{g}$ and $L_{co}$ are the electrodiffusion coefficients of ion transport for counter and co-ions. Their values are found using the following relations:

(6) $$\begin{array}{l} L_{g}=\frac{\kappa_{m}^{DC}}{2F^{2}}\left(1+\sqrt{1-\frac{2F^{2}}{RT}\frac{P^{*}c}{\kappa_{m}^{DC}\pi_{\pm }}}\right)\\ L_{co}=\frac{\kappa_{m}^{DC}}{2F^{2}}\left(1-\sqrt{1-\frac{2F^{2}}{RT}\frac{P^{*}c}{\kappa_{m}^{DC}\pi_{\pm }}}\right) \end{array}$$

where *c* is the concentration of the external solution, mol/m^3^; $\pi_{\pm }$ is the correction factor for the nonideality of the solution.

The correction factor for the nonideality of the solution ($\pi_{\pm }$):

(7) $$\pi_{\pm }=1+\frac{d\ln\gamma_{\pm }}{d\ln c},$$

where $\gamma_{\pm }$ is the average ionic activity ratio of the electrolyte.

The co-ion transport number ($t_{co}^{*}$) can be found as:

(8) $$t_{co}^{*}=1-t_{g}^{*}.$$

The algorithm for calculation of transport numbers using the microheterogeneous model can be found in the Supplementary Information.

## 3. Materials and Methods

### 3.1. Membranes

The objects of the study were heterogeneous ion-exchange membranes MK-40 and MA-41 (Shchekinoazot LLC, Shchekinoazot, Russia) and Ralex CM and Ralex AMH (Mega a.s., Stráž pod Ralskem, Czech Republic). Two samples of each membrane were studied, representing membranes of different batches and years of production (named batch 1 and batch 2 later in the text). Among many heterogeneous ion-exchange membranes, the studied membranes are most common in the southern Russian region.

All studied heterogeneous membranes were produced by hot pressing (MK-40, MA-41) or rolling (Ralex CM, Ralex AMH) of the thermoplastic mixture consisting of the fine powder ion-exchanger and polyethylene, in the approximate ratio of 2:1. The ion-exchanger used in the production of membranes MK-40, MA-41, Ralex CM, and Ralex AMH can be classified as polymer obtained by copolymerization of polystyrene with divinylbenzene. By type of the ionogenic groups, the membranes MK-40 and Ralex CM are strong-acid cation-exchange with sulfonic acid ionogenic groups; MA-41 and Ralex AMH are strong-basic anion-exchange with quaternary ammonium bases.

Physic-chemical properties of the membranes provided by manufacturers are given in Table 2.

All membranes were subjected to the following pre-treatment procedure before the study:

- surface treatment with carbon tetrachloride for degreasing;
- soaking in ethanol for 6 h to remove residues of monomers and oligomers from the ion-exchange resin;
- soaking of the membrane in excess volume (≈20 volumes of the membrane) of 1 M NaCl solution for 24 h;
- washing of the obtained membranes with deionized water to a constant value of the electrical conductivity of the wash water.

The membranes prepared by this method were equilibrated with the working solution in which they were stored before the testing.

### 3.2. Study of the Electrical Conductivity

**Figure 3:**
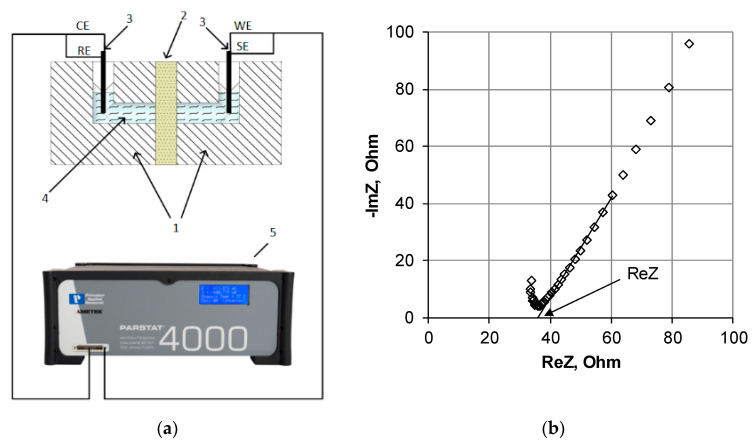
The scheme of the mercury-contact cell (**a**) and a frequency spectrum example of the electrochemical impedance (**b**). 1—mercury-contact cell, 2—studied membrane, 3—platinum electrodes, 4—mercury, and 5–impedance meter.

The electrical conductivity was measured by the electrochemical impedance method using a mercury contact cell [31]. The general view of the measuring cell and the impedance spectrum of the ion-exchange membrane is shown in Figure 3. The method used is one of the contact methods in which the membrane under study is placed between two electrodes (in this method, the electrodes are mercury), after which the impedance of the system is measured. Since mercury impedance is a few hundredths of an ohm and has no reactive component, the measured spectrum of the electrochemical impedance is entirely related to the ion-exchange membrane. In [32], the authors showed that the presence of a solution film on the membrane surface when measuring the resistance by the contact method could lead to underestimated values of electrical conductivity. In this regard, in this work, before measuring the resistance, a film of the solution was removed from the membrane surface using filter paper.

The impedance measurement was carried out using a potentiostat/galvanostat/impedancemeter PARSTAT 4000 (Figure 3a) in the frequency range from 500 kHz to 10 Hz with zero DC component. The amplitude of the AC signal was 100 µA. The ion-exchange membrane’s ohmic resistance (*R*) was found by extrapolating the straight line in the mid-frequency region to the real resistance axis (Figure 3b).

The obtained value is converted into electrical conductivity according to the equation:

(9) $$\kappa_{m}^{AC}=\frac{d}{R_{m}S},$$

where d is the membrane thickness, m; $R_{m}$ is the measured resistance of the membrane, Ohm; *S* is the membrane area, m^2^.

The obtained value of electrical conductivity is called electrical conductivity in the alternating current; it is related to electrical conductivity in direct current ($\kappa_{m}^{DC}$) by the following relation [21] for CEM:

(10) $$\kappa_{m}^{DC}=\kappa_{m}^{AC}{\left(t^{+}\right)}^{f_{2}},$$

and for AEM:

(11) $$\kappa_{m}^{DC}=\kappa_{m}^{AC}{\left(t^{-}\right)}^{f_{2}}.$$

All measurements were carried out at least at five different points to reduce the measurement error. At each point, three spectra were recorded. Then, the mean value was found to calculate the resistance of each sample. The measurements were carried out in the order of increasing the concentration of the equilibrium solution. As a result, the maximum standard deviation in each batch was close to 5% for solutions with the lowest concentration and gradually decreased with an increase of the solution concentration.

### 3.3. Study of the Diffusion Permeability

**Figure 4:**
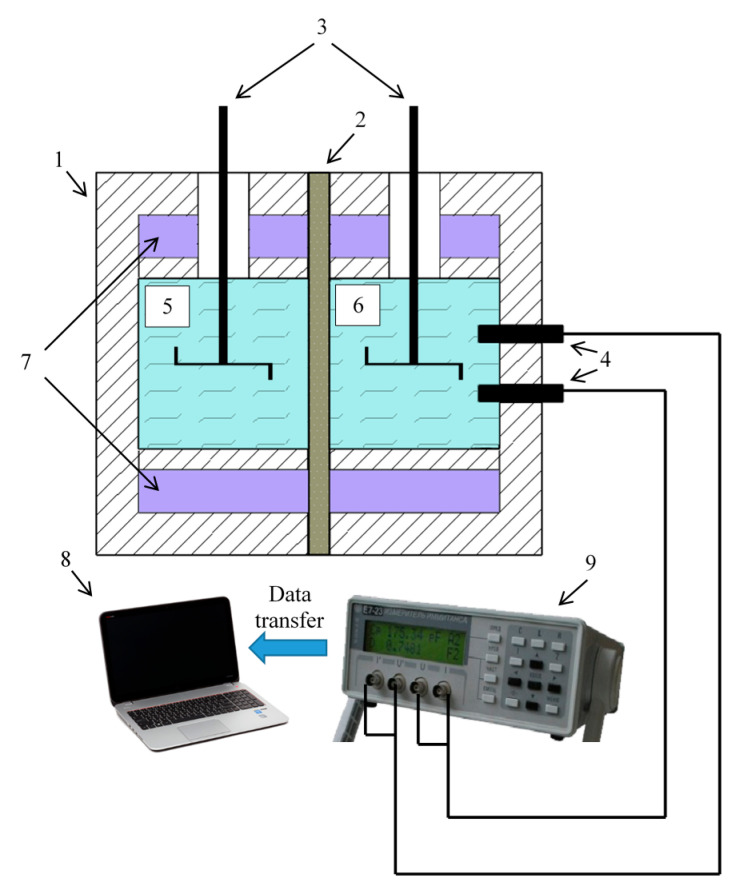
Diffusion cell diagram. 1—Diffusion cell, 2—studied membrane, 3—mechanical mixers, 4—platinum electrodes, 5—half-cell containing salt solution (E chamber), 6—half-cell containing deionized water (DI chamber), 7—water jacket, 8—notebook, 9—E7-21 immittance meter.

The diffusion permeability measurements were carried out in a non-flowing two-chamber cell (Figure 4). The cell consists of two chambers, each with a volume of approximately 100 mL. A conductometric sensor is in one chamber, which registers the change in the solution’s conductivity. This chamber is filled with deionized water, into which the electrolyte diffuses through the membrane (DI chamber). The second chamber, separated from the DI chamber by the investigated ion-exchange membrane, is filled with an electrolyte solution (E chamber). The sodium chloride with different concentrations was used as the electrolyte solution in this work.

Vertical stirrers are placed in both chambers of the cell to diminish the contribution of diffusion boundary layers to the electrolyte transfer rate through the membrane. The stirrer rotation speed is 800 rpm. The distilled water resistance was measured during the experiment at a frequency of 1/20 s using Pt/Pt electrodes connected to the E7-21 immittance meter (MNIPI, Minsk, Bearus).

A blank experiment is carried out before the main measurement to establish the concentration profiles inside the membrane corresponding to the experimental concentration difference. First, the cell is filled with working solutions, and the diffusion process takes place within an hour without registering conductometric data. Then, the solutions in both chambers are replaced, and the diffusion process is repeated with data recording for one hour. Finally, based on the calibration dependence, a graph of the dependence of the electrolyte concentration in the distillate chamber on time is plotted.

The experiment is repeated twice with each test sample to improve the measurement accuracy. On the obtained dependence, the results obtained in the first and last 10 min are discarded. The data for the first 10 min can contain any uncertainty in the flux due to solution replacement. The data for the last 10 min of the experiment are discarded because, for them, the condition of constant concentration difference between compartments (which is strictly imposed by non-equilibrium thermodynamics [33]) is satisfied to a lesser extent than for the rest of the kinetic curve. From the remaining data, the rate of concentration change ($\frac{dc}{d\tau }$) is found. Based on the obtained data, it is possible to calculate the salt flux through the membrane (*j_d_*) and the integral coefficient of diffusion permeability (*P_m_*).

(12) $$j_{d}=\frac{V}{S}\frac{dc}{d\tau },$$

(13) $$P_{m}=\frac{j_{m}d}{c},$$

where *V* is the volume of the deionized water in the DI chamber, L.

### 3.4. Reverse Electrodialysis Experiment

In our study, we used a lab-scale reverse electrodialysis module. The dimensions of the compartments were 5 cm width and 20 cm length, giving an active area of the membrane of 1 dm^2^. The total area of the membrane, including areas which are covered by gaskets, was 8 × 30 cm^2^. Two membrane stacks were studied, one composed of MK-40/MA-41 membranes and another one of Ralex CM/Ralex AMH membranes. The membrane stack was composed of 16 membrane pairs. The membranes were separated with gaskets made of high-density polyethylene. The thickness of the gaskets (*h*) was 0.9 mm both for high and low concentration compartments. The compartments were filled with nonwoven polyethylene spacers (porosity factor of the spacer 0.85). The spacer grid consists of 4 mm squares rotated at 45 degrees to the solution flow.

For the electrode rinse, we used a mixed solution of 0.025 M K_4_[Fe(CN)_6_] and 0.025 M K_3_[Fe(CN)_6_] in 0.25 M NaCl. The electrodes were made from platinum-coated titanium. The iron (III) complex is reduced on the cathode, and the iron (II) complex is reoxidized on the anode. Because the electrode rinse is recirculated through both electrode compartments, the original Fe (III)/Fe (II) ratio is maintained, and there is no net chemical reaction. The cation-exchange membranes were placed near the electrodes to prevent ferri-/ferrocyanide anions migration.

The high concentration solution was modeling the Black Sea, the concentration of which in terms of sodium chloride is 20 g/L, and the low concentration solution was modeling the “river water” with a concentration of 0.2–2.0 g/L NaCl. The solutions in both salt compartments were fed into the module in a one-pass through mode.

The working solutions were pumped through the reverse electrodialysis module from the bottom to the top in a co-flow mode using peristaltic pumps. The volume flow rate was set to 20 L/h for both high and low-concentration compartments. Given the dimensions and number of the compartments (compartment width 5 cm, compartment height 0.9 mm, 16 pair cells) and the porosity of the spacer (0.85), we arrive at a linear velocity of 0.8 cm/s. In some experiments, the flow rate was varied to see how it affects the power output.

All tests were carried out at an ambient temperature of 23 °C.

To measure the power density of the RED module, the external load resistance with ability to change resistance in wide range was connected between cathode and anode in series with multimeter Agilent U1251B (Agilent Technologies, Santa Clara CA, USA) to measure the current. Another multimeter Agilent U1251B was connected parallel to the RED module to measure the voltage in the circuit.

To measure the open circuit voltage of the RED module, the external load was removed from the circuit, and the potential drop between cathode and anode was measured using Ionomer I-130 (Gomel, Soviet Union) with internal resistance 10^9^ Ohms.

## 4. Results and Discussion

### 4.1. Conductivity Measurement Results

**Figure 5:**
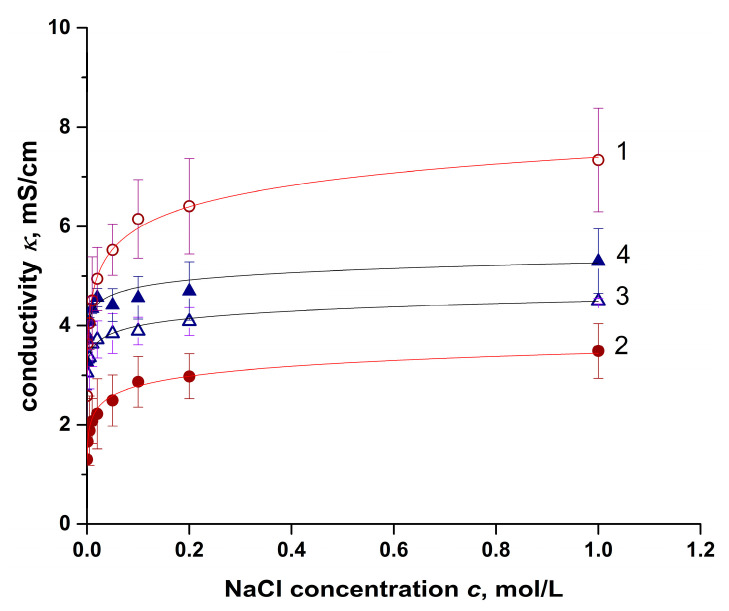
Concentration dependence of the mean electrical conductivity of the ion-exchange membranes from both batches. Membranes: 1—MK-40, 2—MA-41, 3—Ralex CM, 4—Ralex AMH.

The results of measuring the electrical conductivity of ion-exchange membranes of two batches in a wide range of sodium chloride concentrations are shown in Figure 5. The results for each batch are available in Supplementary File S1. The data for each membrane are used for the calculation of the electroneutral solution fraction. It is notable that while the standard deviation is less than 5% at maximum for each batch, for each membrane between two batches, it increases drastically.

Within the microheterogeneous model, the conductivity of a heterogeneous ion-exchange membrane is the geometric mean of the conductivities of the gel phase and the phase of an electrically neutral solution. Given the above, the conductivity of the membrane is expressed as follows:

(14) κm=f1κisoA+f2κsA1A,

where $\kappa_{m}$, $\kappa_{iso}$, and $\kappa_{s}$ are the electrical conductivity of the membrane, gel, and electroneutral solution solution, S/cm; *A* is the characteristic parameter that describes the spatial distribution of conducting phases in the membrane. *A* = +1 for parallel and *A* = –1 for series connected phases; in real samples, the *A* parameter takes values in range 0.1–0.3.

In dilute solutions near the point of isoelectric conductivity (such a value of electrical conductivity when $\kappa_{m}=\kappa_{s}^{}=\kappa_{iso}^{}$), Equation (13) is simplified, and its linearization in bilogarithmic coordinates $\mathrm{lg}\kappa_{m}=f(\mathrm{lg}\kappa_{s}^{})$ makes it possible to determine the value of the parameter *f*_2_.

The found values of the parameter *f*_2_ and the coordinates of the isoelectric conductivity point for the membranes under study are given in Table 3.

It can be seen that for heterogeneous Ralex membranes, the value of the f2 parameter is comparable to the value of this parameter for homogeneous membranes. Such values of this parameter were obtained in other works [34,35,36]. In [34], the authors suggested that such a value of the fraction of the intergel solution for these membranes is a consequence of the fact that the particles of the ion exchanger in these membranes are small enough and, at the same time, there are no macroscopic cavities inside the membrane that electrically neutral solutions can occupy. These structural features of Ralex membranes were demonstrated by Akberova et al. [37] and Slouka et al. [38].

From the perspective of the RED system, a large value of the *f*_2_ parameter (MK-40, MA-41 membranes) can provide high electrical conductivity. This is especially significant for the MK-40 membrane, which has a mean electrical conductivity almost twice as high as the Ralex CM membrane (Figure 5). At the same time, the electrically neutral solution in the membrane’s pores causes a decrease in permselectivity.

Another factor that attracts attention is the large scatter of electrical conductivity values among the samples under study (Figure 5). While for Ralex membranes, the standard deviation values are lower, which suggests better repeatability of the production process, the values of the *f*_2_ parameter for both Ralex CM and Ralex AMH membranes differ almost two-fold. The reason for this can be that different production routes are used for the preparation of different batches. For example, more prolonged milling of the ion-exchange resin results in smaller particles and a higher fraction of macropores in the Ralex CM membrane, as shown in [37].

Regarding MK-40 and MA-41 membranes, a large scatter of their conductivity is also found in the literature, and Veerman gives a good collection of data in [39]. As in the case of Ralex membranes, different production conditions seem to be the leading cause of such a wide range of electrical conductivity. According to Veerman, commercial membranes are not chemical compounds with unchanging properties; different lot numbers, years of production, and storage conditions can significantly influence their properties [39].

### 4.2. Diffusion Permeability Results

As already mentioned in the introduction, the membrane potential is significantly influenced by the permselectivity of the membranes. Diffusion permeability is a value that characterizes the non-selective flux of electrolyte through an ion-exchange membrane.

**Figure 6:**
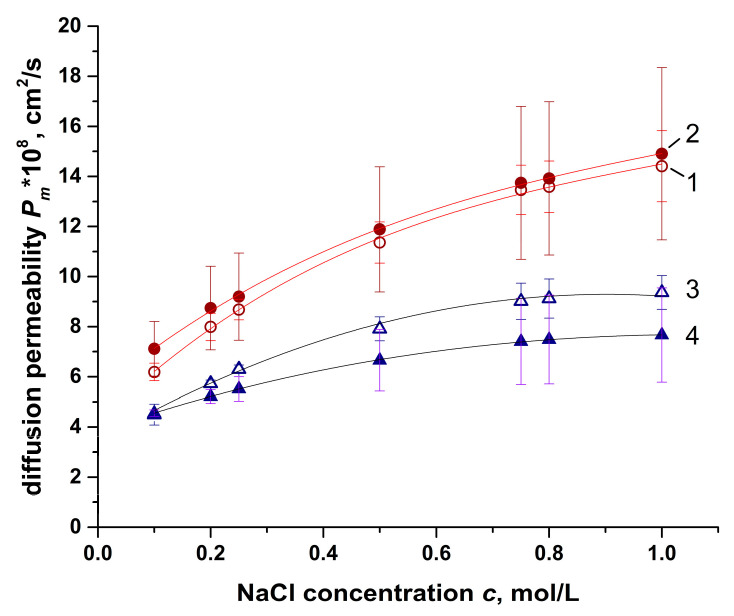
Concentration dependence of the mean diffusion permeability of the ion-exchange membranes from both batches. Membranes: 1—MK-40, 2—MA-41, 3—Ralex CM, 4—Ralex AMH.

The results of measuring the diffusion permeability of the studied ion-exchange membranes in 0.1–1 mol/L NaCl solutions are shown in Figure 6. The results for each batch are available in Supplementary File S1. The data for each membrane are used for the calculation of the *β_j_* parameter.

The results obtained when measuring the diffusion permeability correlate well with the results obtained when studying the electrical conductivity. For Ralex membranes, low values of the *f*_2_ parameter are characteristic, which is reflected in the low dependence of the integral coefficient of diffusion permeability on the concentration of the external solution. At the same time, for the second batch, despite the same low value of the *f*_2_ parameter, the diffusion permeability is comparable to the diffusion permeability of MK-40 and MA-41 membranes. At the same time, no such dependence was revealed for MK-40 and MA-41 membranes. Thus, despite the different values of the integral coefficients of diffusion permeability obtained for different samples, their relationship does not change significantly.

The differential (also sometimes called “local” [36]) diffusion permeability coefficient is used to calculate the transport numbers according to Equation (6). In contrast to the experimentally determined integral coefficient of diffusion permeability, which is the average value over the entire thickness of the ion-exchange membrane, the differential coefficient corresponds to the diffusion permeability of a thin ion-exchange film in equilibrium with a “virtual solution” with a particular concentration *c* at a point in space *x*. The following transformation is used:

(15) $$P^{*}=P_{m}+c\frac{dP_{m}}{dc}=P_{m}\beta_{j},$$

where $\beta_{j}=\frac{d\mathrm{lg}j_{m}}{d\mathrm{lg}c}$ is the parameter that characterizes the concentration profile in the ion-exchange membrane [23] (linear at $\beta_{j}=1$, convex at $\beta_{j}>1$, or concave at $\beta_{j}<1$).

The parameter values found based on experimental data are shown in Table 4.

### 4.3. Transport Numbers

According to the microheterogeneous model, the transport numbers are determined by the combined action of two factors: the electrical conductivity of the membrane (which is mainly determined by the counterion transport) and its diffusion permeability (which is determined by the co-ion transport). Moreover, both of these parameters depend on each other [23]. Thus, membranes with high diffusion permeability are characterized by high electrical conductivity in concentrated solutions. The counterion transfer number for membranes with higher diffusion permeability will be lower, since high diffusion permeability means more co-ions are present in the membrane phase. High electrical conductivity, especially in dilute solutions, is an essential characteristic for the reverse electrodialyzer process, since it reduces the internal resistance of the electromembrane stack.

Counterion transport numbers were calculated using Equation (5). The results are presented in Table 5.

It can be seen that for different samples of Ralex membranes that despite the differences in electrical conductivity and diffusion permeability, the transport numbers differ insignificantly, both for cation-exchange and anion-exchange membranes. For example, the maximum difference in concentrated (1 mol/L) solutions is ±0.01 for Ralex AMH.

For MK-40 and MA-41 membranes, the situation is different. For MK-40 membrane, the difference in transport numbers reaches ±0.05. On the other hand, the largest scatter was obtained for the MA-41 membrane, where, depending on the batch, the difference is ±0.1.

### 4.4. OCV and Power Density of the RED Stack

**Figure 7:**
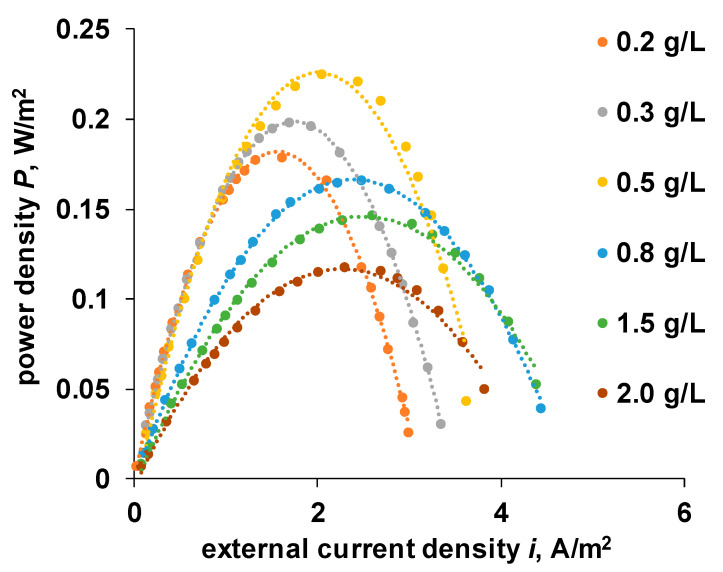
Dependence of power density generated by reverse electrodialysis module on external current density and salinity of the low concentration solution. Membrane pair: Ralex CM/Ralex AMH.

The raw data of the dependence of the power density on the current density in the external circuit are shown in Figure 7.

**Figure 8:**
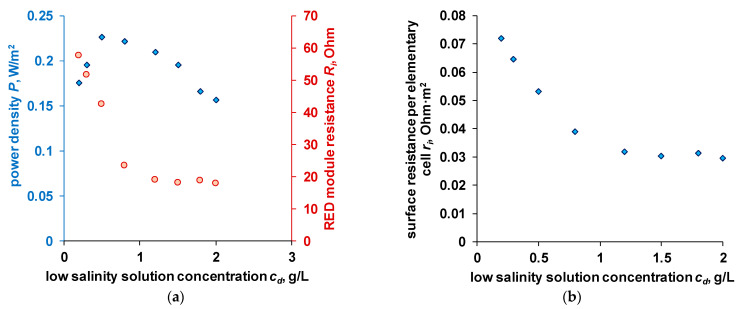
Dependence of power density and RED module resistance (**a**) and surface resistance of one elementary cell (**b**) on the salinity of the low concentration solution. Membrane pair: Ralex CM/Ralex AMH.

It is known that the maximum power can be obtained when the resistance of the external load is equal to the internal resistance of the reverse electrodialysis module [40]. The power density and internal resistance obtained on a laboratory reverse electrodialysis module are shown in Figure 8.

The total inner resistance of the reverse electrodialyzer membrane stack (*R_i_*) can be divided into several components:

(16) $$R_{i}=R_{\mathrm{Ohm}}+R_{n/\mathrm{Ohm}}+R_{\mathrm{el}}.$$

where $R_{\mathrm{Ohm}}$, $R_{n/\mathrm{Ohm}}$, and $R_{\mathrm{el}}$ are the ohmic and non-ohmic components of the resistance of the membrane stack and the resistance of the solution in the electrode chambers, Ohm.

The resistance of the solution in the electrode chambers can be measured directly.

The ohmic component of the resistance includes the resistances of ion-exchange membranes and high and low salinity solutions. Thus, for the entire membrane stack, the resistance can be found as:

(17) $$R_{\mathrm{Ohm}}=\frac{N}{S}\left(\frac{d_{\mathrm{CEM}}}{\kappa_{\mathrm{CEM}}^{DC}}+\frac{d_{\mathrm{AEM}}}{\kappa_{\mathrm{AEM}}^{DC}}+\frac{h_{b}}{\kappa_{b}}+\frac{h_{d}}{\kappa_{d}}\right)=\frac{N}{S}\left(R_{\mathrm{CEM}}^{s}+R_{\mathrm{AEM}}^{s}+R_{b}^{s}+R_{d}^{s}\right),$$

where *h* is the thickness of the gasket, m; superscript «*s*» marks the surface resistance in Ohm·m^2^. The conductivity of the solutions in the low and high concentration chambers must take into account the porosity factor of the spacer.

Non-ohmic components of resistance include the change in resistance along the channel length associated with a change in concentration and the contribution of the resistance of diffusion boundary layers at the membrane/solution interface:

(18) $$R_{n/\mathrm{Ohm}}=N\left(\left(R_{\Delta c}^{d}+R_{\Delta c}^{b}\right)+2\left(R_{\mathrm{EBL}}+R_{\mathrm{DBL}}\right)\right)=N\left(R_{\Delta c}^{*}+R_{\mathrm{BL}}^{*}\right).$$

The first term in parentheses is responsible for the change in resistance along the length of the channel with high and low salinity solutions. The second term represents the change in resistance in the enriched (from the side of the channel with a low salinity solution) and depleted (from the side of the channel with high salinity solution) diffusion layers.

In the general case, the non-ohmic components of the resistance of the membrane stack of an electrodialyzer are neglected based on the following considerations:

1. The change in the concentration of the solution with a not very long channel length is insignificant. Considering the values of the integral diffusion permeability coefficient given in Section 4.2 and the linear velocity of the solution, and the geometric parameters of the electrodialyzer, the calculated concentration decrease in the high salinity solution is 0.002 mol/L. Such changes will not have a significant impact on resistance.
2. The resistance of diffusion layers is also generally not considered. In the case of an enriched diffusion layer ($R_{\mathrm{EBL}}$), the concentration at the membrane surface is higher than in the bulk of the solution, and its resistance will be lower. In the case of a depleted diffusion layer ($R_{\mathrm{DBL}}$), it is assumed that a separator is sufficiently effective, so the thickness of this layer is sufficiently small. The decrease of concentration in the depleted diffusion layer against the bulk of a high salinity solution is also assumed to be insignificant. The dependence of power density on the linear velocity of the solution in the high salinity channel verifies the later statement (see Supplementary).

For comparison of electrodialysis modules of different designs, it is convenient to use specific values. The surface resistance per unit cell can be calculated as:

(19) $$r_{i}=\frac{S}{N}\left(R_{i}-R_{\mathrm{el}}\right).$$

When considering the dependence of the resistance of the RED module on the concentration of a low salinity solution (Figure 8), two sections can be distinguished: the first corresponds to a sharp drop in resistance as the concentration of the solution entering the low concentration compartment increases (conditional river water). A sharp drop in resistance is observed up to a solution concentration of 0.8 g/L. Further increase in the low salinity solution concentration does not significantly decrease the RED module resistance.

This nature of the dependence is associated with a change in the “limiting phase”, the material with the maximum resistance in the membrane stack. In solutions with a low concentration, ion-exchange membranes have higher conductivity, while the electrical conductivity is linearly dependent on its concentration. As the concentration of the solution approaches the concentration of *c*_iso_, the contributions to the total conductivity of the ion-exchange membranes and the low salinity solution become comparable (Table 6). Obviously, with a further increase in concentration and when *c*_iso_ is exceeded, the resistance of the membrane stack will remain practically constant.

As can be seen from the table, the main contribution to the resistance of the membrane stack in dilute solutions is made by the solution in the low salinity solution compartment. When using solutions with a higher concentration, the total contribution of ion-exchange membranes becomes more significant than the solution’s contribution. In this case, the resistance of the solution in the path with high salinity solution and the solution of the electrode chambers does not significantly affect the total ohmic resistance of the apparatus.

The maximum power density ($P_{\max}^{s}$) obtained in the process of reverse electrodialysis depends on two parameters [41]: the open circuit potential and the internal resistance of the apparatus.

(20) $$P_{\max}^{s}=\frac{{\left(\mathrm{OCV}\right)}^{2}}{4R_{i}S}.$$

**Figure 9:**
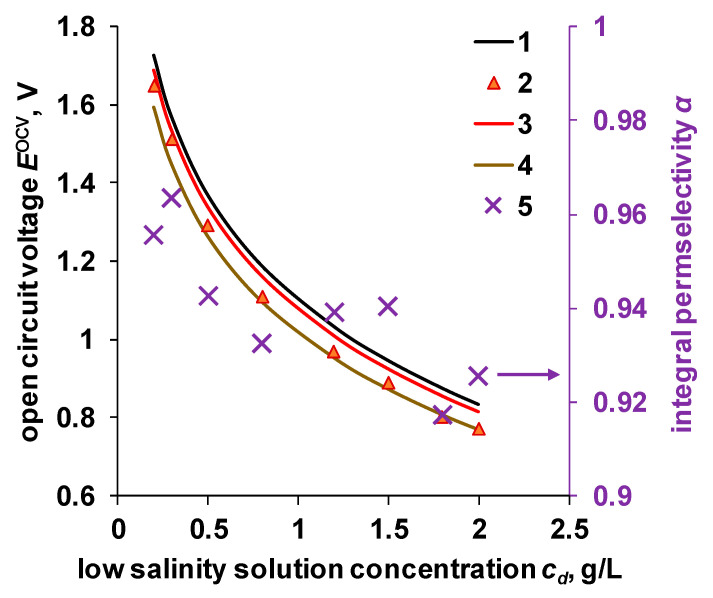
Dependence of the OCV on the RED module on low salinity solution concentration. 1—theoretical value calculated using Equation (1) with *α* = 1, 2—experimental OCV value for Ralex membrane pair, 3 and 4—theoretical value calculated for Ralex and MK-40/MA-41 membrane pairs using Equations (2) and (5), 5—integral permselectivity for Ralex membrane pair.

As already indicated, the OCV value can be established knowing the characteristics of the membranes using Equations (2) and (5). The calculation results, as well as experimental data, are shown in Figure 9. Due to the large scatter of the experimental data (see Supplementary) and to minimize uncertainty in the characteristics, we used the average values between the test results of two batches for calculation of the OCV and power characteristics of the RED module.

From the results shown in Figure 9, it can be seen that the calculated OCV value for the Ralex membrane pair is close to the ideally selective membrane pair, for which α = 1. Low selectivity of the MK-40 and MA-41 membranes negatively affects the open circuit potential (OCV). The experimentally observed OCV values for the Ralex membrane pair are lower than the theoretical ones and are close to the values calculated for the MK-40/MA-41 membrane pair. The deviation of the experimental values from the theoretical ones can be explained by the difference between the membrane samples, the presence of assembly defects in the electrodialysis module, and leakage currents.

Based on the obtained experimental data, the integral permselectivity of the Ralex membrane pair was calculated. The calculation was carried out similarly to the calculation of the apparent transfer numbers obtained by measuring the membrane potential, as the ratio of the experimental potential to the theoretical one:

(21) $$\alpha =\frac{E_{\exp}^{OCV}}{E_{theor}^{OCV}}=\frac{1}{2}\left(\alpha^{\mathrm{CEM}}+\alpha^{\mathrm{AEM}}\right),$$

where $E_{\exp}^{\mathrm{OCV}}$ is the experimental open circuit potential, V; $E_{theor}^{\mathrm{OCV}}$ is the theoretical open circuit potential, calculated using Equation (1) with *α* = 1, V.

The resulting integral permselectivity is essentially an average value that considers all those changes in the solution concentration along the length of the electrodialyzer channel, the dispersion of the membrane characteristics over the sample area, and the number of membranes of each type. The mean value found for the Ralex membrane pair in the investigated concentration range of the low salinity solution was 0.94.

**Figure 10:**
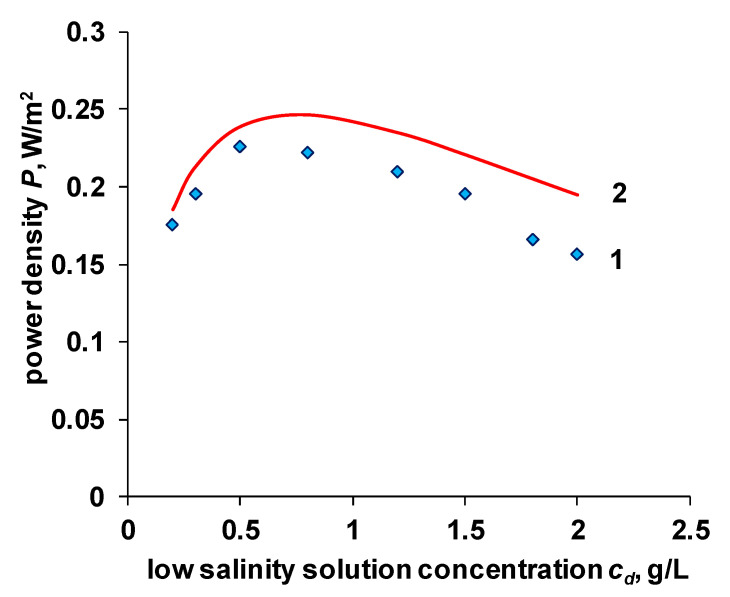
Dependence of power density on the salinity of the low concentration solution. 1—experimental data for the Ralex membrane pair, 2—calculation using Equation (20).

The dependences of the electrical conductivity of ion-exchange membranes on the concentration of the external solution obtained in this work, together with the theoretically calculated values of the OCV potential, can be used to calculate the theoretical value of the power density (Equation (19)). The calculation results and their comparison with experimental data are presented in Figure 10.

The calculated values of the power density are higher than the experimental results. This can be explained by the fact that when calculating according to Equation (20), the internal resistance of the membrane package does not consider the non-ohmic components of the resistance. Another possible reason can be the spacer shadow effect [42]. The significant difference between the theoretical and experimental values observed at concentrations of a diluted solution above 0.5 g/L can be associated with both the underestimated value of the internal resistance and the underestimated values of the permselectivity of ion-exchange membranes in these solutions (Figure 9).

Another possible explanation for the decrease in power density is the appearance of parasitic currents or leakage currents. This phenomenon is well known for the electrodialysis process, especially in the field of concentrated solutions [43,44] or bipolar electrodialysis [45]. Some of the current generated by the reverse electrodialyzer tends to flow through a common collector with brine solution. This is because the electrical resistance of such a collector is significantly lower than the resistance of the rest of the components of the system and because it provides an almost direct path of current flow from the cathode to the anode. Mathematical modeling and experimental studies carried out in [40,46] showed that the share of power losses from the occurrence of parasitic currents increases with an increase in the number of pair cells and can reach 30–40%.

Let us consider the possible share of power losses associated with parasitic currents in this work. The investigated reverse electrodialyzer has a sufficiently large number of pair cells (*N* = 16). Based on the literature data, this is approximately the lower limit at which the effects of parasitic currents begin to build. However, the share of power losses, in this case, does not exceed 5% (calculated value from [40]) and is even less in the experiment. In the above-mentioned studies, the concentration of the brine solution was 5 M, which is much higher than the solution of 20 g/L (0.34 M) used in this work.

In addition, the investigated RED module has an elongated channel shape (the length-to-width ratio of the channel is 4:1), which makes it possible to use only one feed and drain channel, which should create additional resistance that prevents the parasitic flow of current. However, this channel geometry can be disadvantageous at low fluid flow rates, since dead zones can appear at the corners of the channel. The flow of the solution in stagnant zones is low, significantly reducing the concentration gradient between high and low concentrations chambers. Indirectly, the small influence of such zones is indicated by the absence of dependence of the power density on the flow through the module. However, to confirm this fact, additional experimental studies are needed.

## 5. Conclusions

This work shows the possibility of using a microheterogeneous model for describing the properties of ion-exchange membranes and calculating the characteristics of a reverse electrodialyzer using the data obtained. We studied the properties of eight samples of heterogeneous ion-exchange membranes (two samples of each type of membrane). The samples differed in the year of issue and storage conditions. It has been shown that for heterogeneous ion-exchange membranes MK-40 and MA-41, the properties of the samples can differ significantly. Both the electrical conductivity (higher for batch 2) and diffusion permeability (lower for batch 2) differ, which ultimately leads to a wide scatter of the obtained values of the transfer numbers of counterions. For Ralex membranes, such significant differences were not observed between different samples, except for the extremely low diffusion permeability of the Ralex AMH membrane (batch 1). The MK-40 membranes are a good choice from the internal resistance point of view, as they show higher conductivity. On the other hand, the Ralex membranes show better permselectivity, which is crucial for the RED process. In addition, the properties of the Ralex membranes are better reproduced between batches, as compared to MK-40 and MA-41 membranes. As such, in the view of the present work, a Ralex membrane pair is preferable for RED.

The possibility of calculating the transfer numbers and predicting the open-circuit potential on this basis will allow in the future selecting the best membrane pairs for the reverse electrodialysis process based on measuring their physicochemical characteristics. The data obtained from these measurements on the electrical conductivity of ion-exchange membranes can also be used to calculate the ohmic components of the internal resistance of the electrodialyzer. The latter characteristic, in turn, allows calculating not only the open circuit potential, but also the theoretical power density.

## Figures and Tables

**Table 1:** Methods used for transport numbers’ determination.

Name	Method	Advantages	Drawbacks	Ref.
Membrane potential	An IEM is placed between solutions with different concentrations of the same ions, and permselectivity (expressed in terms of the ion-transport number) is obtained from the electrochemical potential difference between the two solutions with high and low concentration: $\begin{array}{l} t_{app}=\frac{1}{2}\left(\frac{E_{m}}{E_{theor}}\right)+1\\ E_{theor}=\frac{RT}{F}\ln\frac{a_{\pm }^{b}}{a_{\pm }^{d}} \end{array}$	Simplicity of measurement	Only apparent transport numbers can be determined. Additionally, the exact ion-transport numbers determined in this way depend on the measurement conditions	[18]
Scatchard	If apparent transport number and water transport numbers are known, the true transport numbers can be calculated: $t_{g}=t_{app}+m_{\pm }M_{w}t_{w}$	True transport numbers can be calculated	Requires the knowledge of concentration dependence of water transport numbers. The measurement of the latter is complicated	[19]
Three-wire model	If parameters of the three-wire model are known, the true transport number can be calculated in assumption that co-ions are transported only through solution channel of conductivity: $t_{g}=1-c\frac{\kappa_{s}}{\kappa_{iso}}$	Requires only concentration dependence of electrical conductivity	In concentrated solutions, when transport of co-ions in the gel becomes significant, tends to predict lower values of transport numbers	[20]
Microheterogeneous model	True counter ions transport number can be calculated based on the concentration dependence of electrical conductivity and diffusion permeability. See text for more information.	Considers transport through the gel and electroneutral solution. True transport numbers can be calculated.	Large amount of experimental data is required.	[21]

**Table 2:** Physic-chemical properties of the membranes studied.

Membrane	Ralex CM	Ralex AMH	MK-40	MA-41
Functional groups	−SO_3_^−^	−N^+^(CH_3_)_3_	−SO_3_^−^	−N^+^(CH_3_)_3_
Counterion in NaCl solution	Na^+^	Cl^–^	Na^+^	Cl^–^
Ion-exchange resin	Lewatit S100	Lewatit M500	KU-2-8	AV-17-8
Inert binder	LDPE			
Reinforcing mesh	Ulester 32S		Nylon	
Ion-exchange capacity, mmol/g-swollen	1.12	0.86	1.08	0.91
Water content, %	44	45	33	36
Surface resistance ^1^, Ohm·cm ^2^	<8	<7.5	<10	<11
Permselectivity ^2^, %	>90	<90	>80	>94
Wet thickness, microns	720	750	540	530

^1^ Measured in 0.5 M NaCl solution; ^2^ measured between 0.5/0.1 M KCl solution.

**Table 3:** Values of coordinates of the point of isoelectric conductivity and transport-structural parameter *f*_2_ for the studied ion-exchange membranes in NaCl solution.

Membrane *	*f*_2_	$\kappa_{iso}^{},\;\mathrm{mS}/\mathrm{cm}$	*c_iso_*, mol/L
MK-40 1	0.10	5.0	0.046
MK-40 2	0.12	6.0	0.056
Ralex CM 1	0.06	3.6	0.032
Ralex CM 2	0.03	4.0	0.037
MA-41 1	0.12	1.9	0.017
MA-41 2	0.14	2.8	0.025
Ralex AMH 1	0.04	4.3	0.039
Ralex AMH 2	0.07	4.6	0.042

* The number near membrane name indicates the batch.

**Table 4:** Parameter $\beta_{j}$ values found for the studied ion-exchange membranes.

Membrane *	MK-40 1	MK-40 2	MA-41 1	MA-41 2	Ralex CM 1	Ralex CM 2	Ralex AMH 1	Ralex AMH 2
$\beta_{j}$	1.39	1.35	1.36	1.26	1.22	1.43	1.02	1.39

* The number near membrane name indicates the batch.

**Table 5:** Calculated counterion transport numbers for the studied ion-exchange membranes.

NaCl Concentration	$\mathrm{Counter}\;\mathrm{Ion}\;\mathrm{Transport}\;\mathrm{Number}\;(t_{g}^{*})\;\mathrm{Calculated}\;\mathrm{Using}\;\mathrm{Equation}\;(5)$							
	MK-40 1	MK-40 2	MA-41 1	MA-41 2	Ralex CM 1	Ralex CM 2	Ralex AMH 1	Ralex AMH 2
0.05	0.99	0.99	0.99	0.99	0.99	0.99	0.99	0.99
0.1	0.99	0.99	0.98	0.99	0.99	0.99	0.99	0.99
0.2	0.99	0.99	0.97	0.99	0.99	0.99	0.99	0.99
0.5	0.96	0.98	0.91	0.97	0.97	0.97	0.98	0.98
0.8	0.93	0.97	0.82	0.95	0.96	0.96	0.98	0.96
1.0	0.91	0.96	0.73	0.94	0.95	0.95	0.97	0.96

* The number near membrane name indicates the batch.

**Table 6:** Contribution of various components to the ohmic resistance of the reverse electrodialyzer membrane stack.

	Low Salinity Solution Concentration, g/L	CEM	AEM	Low Salinity	High Salinity	Electrode Solution
Fraction of ohmic resistance ($R_{j}/\left(R_{\mathrm{Ohm}}+R_{\mathrm{el}}\right)$)	0.5	0.14	0.11	0.71	0.02	0.02
	2.0	0.29	0.21	0.40	0.05	0.04

## Supplementary Materials

The following are available online at https://www.mdpi.com/article/10.3390/membranes11060406/s1. Algorithm for calculation of transport numbers, RED stack OCV and power density. S2. Additional figures. B1. Concentration dependence of the electrical conductivity of the ion-exchange membranes batch 1 and batch 2. B2. Concentration dependence of the diffusion permeability of the ion-exchange membranes batch 1 and batch 2. B3. Concentration dependence of the counterion transport number for cation-exchange and anion-exchange membranes. B4. Dependence of power density on solution velocity.

## List of Symbols and Abbreviations

*Subscripts and superscripts*:		
Subscript “*b*” denotes high concentration solution (brine),		
Subscript “*d*” denotes low concentration solution (diluate),		
Subscript CEM shows that characteristic is for cation-exchange membrane,		
Subscript AEM shows that characteristic is for anion-exchange membrane,		
Superscript “*s*” denotes that resistance is in Ohm·m^2^.		
*Abbreviations*:		
CEM	cation exchange membrane	
AEM	anion exchange membrane	
RED	reverse electrodialysis	
OCV	open circuit voltage	
*Greek letters*:		
Parameter	Description	Dimension
α	permselectivity of ion-exchange membrane	
*A*	characteristic parameter that describes the spatial distribution of conducting phases in the membrane	
*β_j_*	parameter that characterizes the concentration profile in the ion-exchange membrane	
*γ* ^+^	cation activity coefficient	
*γ* ^–^	anion activity coefficient	
*γ* ^±^	mean activity coefficient of electrolyte	
$\kappa_{s}^{DC}$	electrical conductivity of membrane on direct current	S/m
$\kappa_{s}^{AC}$	electrical conductivity of membrane on alternating current	S/m
$\kappa_{m}$	the specific electrical conductivity of the membrane	S/m
$\kappa_{s}^{}$	the specific electrical conductivity of the electrolyte	S/m
$\kappa_{iso}^{}$	the specific electrical conductivity of the gel phase	S/m
$\pi_{\pm }$	correction factor for the nonideality of the solution	
*τ*	time	s
*English letters*:		
Parameter	Description	Dimension
*c*	electrolyte concentration	mol/L
*d*	membrane thickness	m
*E* ^OCV^	open circuit voltage	V
*E_m_*	membrane potential	V
*E*_RED_	potential generated on the membrane stack	V
$E_{\exp}^{\mathrm{OCV}}$	experimental open circuit potential	V
$E_{theor}^{\mathrm{OCV}}$	theoretical open circuit potential	V
*f*_1_, *f*_2_	volume fractions of the gel and electroneutral solution phases	
*F*	Faraday’s constant	C/mol
*h*	solution compartment thickness	m
*i*	current density	A/m^2^
*j_d_*	diffusion salt flux through the membrane	mol/(m^2^·s)
*J*_i_	flux of ion i in the membrane	mol/(m^2^·s)
*L*_g_	counterion electrodiffusion coefficient	
*L*_co_	co-ion electrodiffusion coefficients	
*n*	number of electrons in RedOx reaction	
*N*	number of membrane pairs (elementary cells)	
*P* ^*^	differential coefficient of diffusion permeability of the membrane	m^2^/s
*P_m_*	integral coefficient of diffusion permeability of the membrane	m^2^/s
$P_{\max}^{s}$	maximum power density obtained in RED	W/m^2^
*R*	universal gas constant	J/(K·mol)
*r_i_*	surface resistance per unit cell	Ohm∙m^2^/N
*R_i_*	internal resistance of the membrane stack	Ohm
*R_m_*	measured resistance of the membrane	Ohm
*R*_Ohm_	membrane stack ohmic component of the resistance	Ohm
*R*_n/Ohm_	membrane stack non-ohmic component of the resistance	Ohm
*R*_el_	resistance of the solution in the electrode chambers	Ohm
*S*	membrane area	m^2^
$t_{g}^{*}$	electromigration transport number of counterions in the membrane	
$t_{co}^{*}$	electromigration transport number of co-ions in the membrane	
*t* ^+^	cation transport number in solution	
*t* ^–^	anion transport number in solution	
*T*	absolute temperature	K
*V*	volume of the solution	m^3^
*V*_gel_	volume of the gel phase	m^3^
*V*_sol_	volume of the electroneutral solution phase and total volume of the membrane	m^3^
*V*_total_	total volume of the membrane	m^3^
*z_i_*	charge of ion *i*	
