# Supplementary material for: Use of the Microheterogeneous Model to Assess the Applicability of Ion-Exchange Membranes in the Process of Generating Electricity from a Concentration Gradient
Source: Membranes (Basel). 2021 May 28;11(6):406. doi: 10.3390/membranes11060406 (PMC8230344; doi:10.3390/membranes11060406)
Supplement: Supplementary file 1 [file membranes-11-00406-s001.zip › membranes-1213669-supplementary.pdf]

The counterion transport number can be found if electrotransport (L_g and L_{co}) coefficients are known:

$$t_g^* = \frac{L_g}{L_g + L_{co}} \tag{A1}$$

$$L_g = \frac{\kappa_m^{DC}}{2F^2}\left(1 + \sqrt{1 - \frac{2F^2}{RT}\frac{P^* c}{\kappa_m^{DC}\pi_\pm}}\right) \tag{A2}$$

$$L_{co} = \frac{\kappa_m^{DC}}{2F^2}\left(1 - \sqrt{1 - \frac{2F^2}{RT}\frac{P^* c}{\kappa_m^{DC}\pi_\pm}}\right) \tag{A3}$$

The equations above allows to calculate the value of t_g^* if electrical conductivity (κ_m^{DC}) and differential diffusion permeability (P^*) are known at a given concentration (c). To calculate the electrotransport coefficient at an arbitrary concentration two dependences are required:

1. Concentration dependence of electrical conductivityon direct current $\kappa_m^{DC} - f(c)$;
2. Concentration dependence of differential coefficient of diffusion permeability $P^* - f(c)$.

However, in experiment we usually obtain values of electrical conductivity on alternating current (κ_m^{AC}) and integral coefficient of diffusion permeability (P_m). To obtain required chatacteristics the following equations are used:

$$\kappa_m^{DC} = \kappa_m^{AC}\left(t_g\right)^{f_2} \tag{A4}$$

$$P^* = P_m \beta_j \tag{A5}$$

Coefficients f_2 and β_j are parameters in the microheterogeneous model:

$$f_2 = \frac{d\lg\kappa_m^{AC}}{d\lg\kappa_s} \tag{A6}$$

$$\beta_j = \frac{d\lg j_m}{d\lg c} \tag{A7}$$

Besides the abovementioned dependencies and parameters some properties of the solution are required: conductivity data, transport numbers of ions in the solution, dependence of mean activity coefficient on concentration to calculate $\pi_\pm$. Table below provides this data for sodium chloride solution.

Electrolyte	Conductivity (κ_s), mS/cm [1]	Na^+ transport number	Cl^- transport number	Non-ideality coefficient ($\pi_\pm$)
NaCl	$\kappa = -1.4 \cdot 10^{-5} + 126.6c - 98.0c^{3/2} - -14.7c^2 \ln c + 105.1c^2 - 120.6c^{5/2} + +126.6c^3 - 73.9c^{7/2} + 22.7c^4 - 2.8c^{9/2}$	0.396	0.604	$\pi_\pm = 0.902 - 7.506 \cdot 10^{-3} c$

When counterions transport numbers are found one can estimate the permselectivity of the cation-exchange and anion-exchange membranes and membrane stack OCV can be calculated:

$$E^{OCV} = N \frac{RT}{nF} \ln\left(\frac{c_b \gamma_b^\pm}{c_d \gamma_d^\pm}\right)\left(\frac{T_{CEM}^+ - t^+}{1 - t^+} + \frac{T_{AEM}^- - t^-}{1 - t^-}\right) \quad \text{(A8)}$$

The maximum power density obtained in the process of reverse electrodialysis depends on two parameters: the open circuit potential and the internal resistance of the RED module.

$$P_{\max}^s = \frac{(\mathrm{OCV})^2}{4 R_i S} \quad \text{(A9)}$$

If the non-ohmic components of the membrane stack resistance are negligible, then the internal resistance of the RED module can be found as:

$$R_i = \frac{N}{S}\left(\frac{d_{\mathrm{CEM}}}{\kappa_{\mathrm{CEM}}^{DC}} + \frac{d_{\mathrm{AEM}}}{\kappa_{\mathrm{AEM}}^{DC}} + \frac{h_{\mathrm{b}}}{\kappa_{\mathrm{b}}} + \frac{h_{\mathrm{d}}}{\kappa_{\mathrm{d}}}\right) + R_{\mathrm{el}} \quad \text{(A10)}$$

Algorithm for calculation of transport numbers, RED stack OCV and power density:

1. The value of the f_2 is determined from the dependence of the specific electrical conductivity of the membrane on the specific electrical conductivity of the equilibrium solution, in the bi-logarithmic coordinates $\lg \kappa_m^{AC} - f(\lg \kappa_s)$. From the value of the tangent of the angle of inclination of the obtained dependence, the parameter f_2 is found (Eq. (A6)).

2. The value of the β parameter is determined from the dependence of the diffusion flux on concentration in bi-logarithmic coordinates $\lg j_m - f(\lg c)$. From the value of the tangent of the angle of inclination of the obtained dependence, the parameter β is found (Eq. (A7)).

3. Using found values and experimental data the electrical conductivity on alternating current (κ_m^{AC}) and integral coefficient of diffusion permeability (P_m) using Eqs. (A4) and (A5).

4. The counterion transport number can now be found in the concentration range at which experimental data is measured, using Eqs. (A1)-(A3).

5. The membrane stack theoretical OCV is calculated using Eq. (A8).

6. The internal resistance of the membrane stack can be calculated using experimental data on electrical conductivity of the membranes and literature data for solution specific conductivity, using Eq. (A10).

7. Using the calculated OCV and internal resistance one can calculate the maximum power density of the RED stack at a given concentration difference with Eq. (A9).

Reference:

1. Wadsworth, J. C. The Statistical Description of Precision Conductivity Data for Aqueous Sodium Chloride. *J. Solution Chem.* **2012**, 41, 715–729. doi: 10.1007/s10953-012-9823-6.

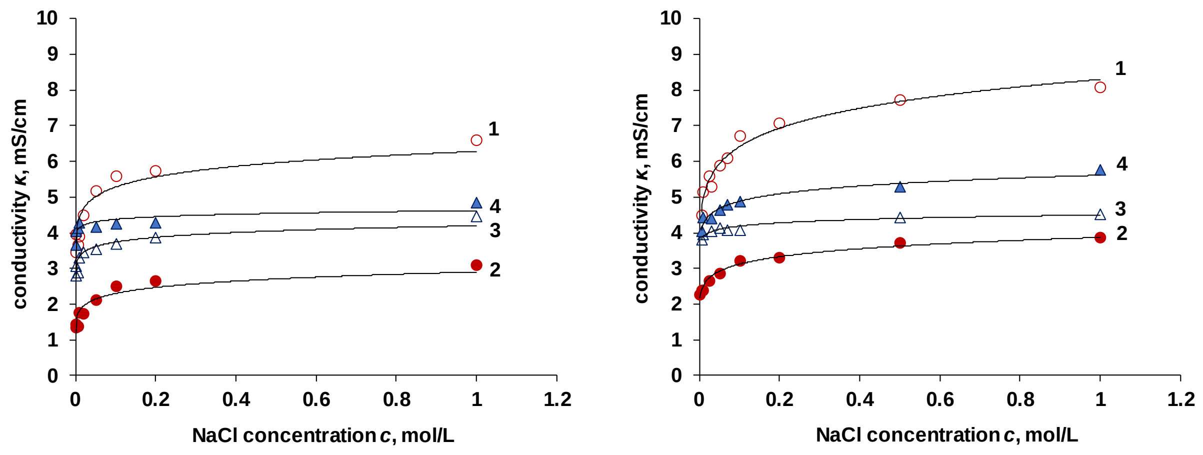

a *b*

Figure B1. Concentration dependence of the electrical conductivity of the ion-exchange membranes batch 1 (a) and batch 2 (b). Membranes: 1 – MK-40, 2 – MA-41, 3 – Ralex CM, 4 – Ralex AMH.

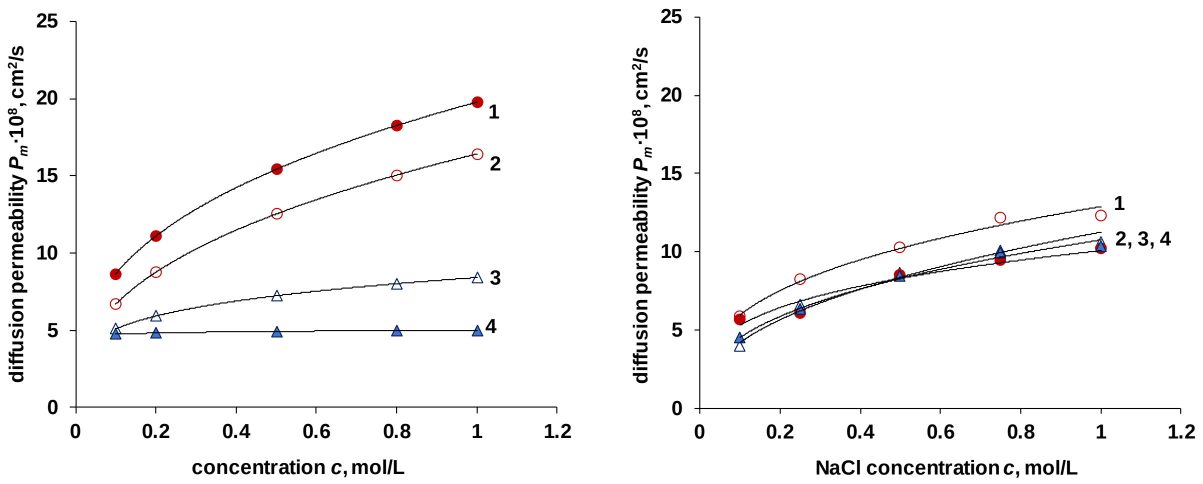

a *b*

Figure B2. Concentration dependence of the diffusion permeability of the ion-exchange membranes batch 1 (a) and batch 2 (b). Membranes: 1 – MK-40, 2 – MA-41, 3 – Ralex CM, 4 – Ralex AMH.

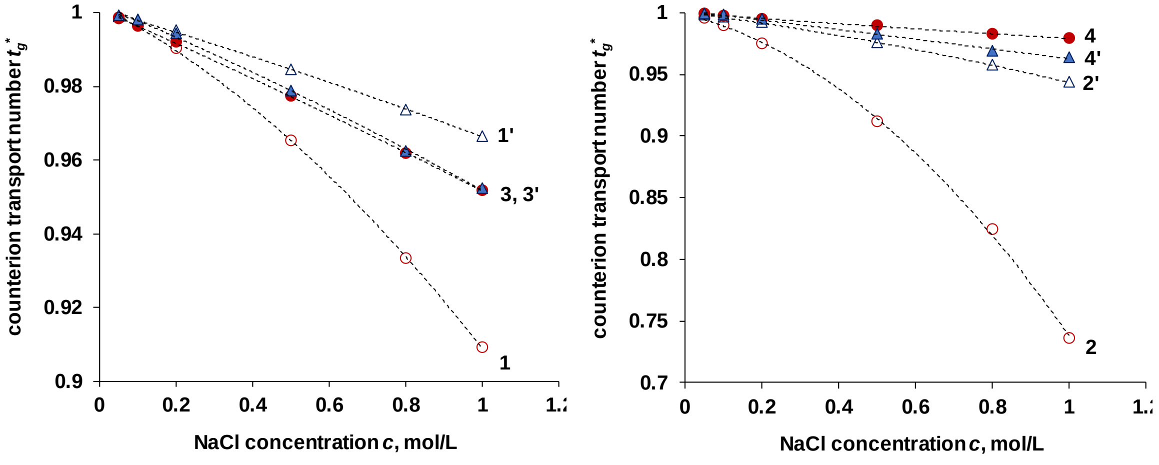

a *b*

Figure B3. Concentration dependence of the counterion transport number for cation-exchange (*a*) and anion-exchange (*b*) membranes. Batch 1 (*a*) and batch 2 (*b*). Membranes: 1, 1′ – MK-40, 2, 2′ – MA-41, 3, 3′ – Ralex CM, 4, 4′ – Ralex AMH. Membranes from batch 1 (1, 2, 3, 4) and membranes from batch 2 (1′, 2′, 3′, 4′).

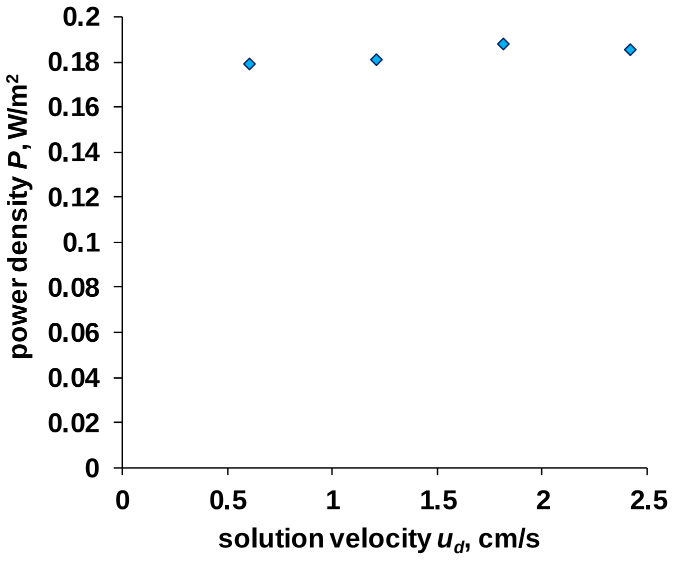

Figure B4. Dependence of power density on solution velocity.
